# Predictors of suicidal behavior in a sample of incarcerated individuals

**DOI:** 10.47626/2237-6089-2024-0821

**Published:** 2025-09-09

**Authors:** Victor H. V. Benvindo, Antônio B. C. Machado, Gabriel D. Curra, Maria V. S. Wingen, Rosa M. M. de Almeida

**Affiliations:** 1 Universidade Federal do Rio Grande do Sul Porto Alegre RS Brazil Programa de Pós-Graduação em Psicologia, Universidade Federal do Rio Grande do Sul, Porto Alegre, RS, Brazil.

**Keywords:** Suicidal ideation, suicide attempted, impulsive behavior, social support, substance-related disorders, prisoners

## Abstract

**Objective:**

Incarcerated individuals exhibit higher suicide rates compared to the general population. Investigating risk factors aids in developing effective public policies and interventions. The goal of this study was to assess and analyze factors predicting both suicidal thoughts and suicide attempts in a population of male incarcerated individuals who engage in the use of multiple psychoactive substances.

**Methods:**

A cross-sectional observational study was conducted. A total of 174 male individuals deprived of liberty participated in the study, all of whom were serving a closed prison sentence during the data collection steps. Participants were assessed with the following instruments: the Addiction Severity Index (ASI-6) and the Barratt Impulsiveness Scale (BIS-11).

**Results:**

Amongst our sample, observed prevalences were 36.7% for suicidal ideation and 16% for suicide attempts. Impulsivity (odds ratio [OR] = 1.098, 95% confidence interval [95%CI] 1.008-1.197), social support (OR = 0.281, 95%CI 0.085-0.925), witnessing someone being killed or beaten (OR = 5.173, 95%CI 2.143-12.486), cigarette use (OR = 3.309, 95%CI 1.063-10.293), and cocaine use (OR = 2.678, 95%CI 1.040-6.897) were found to be associated with suicidal ideation. No significant associations were found between drug use and suicide attempts.

**Conclusion:**

A high prevalence of suicidal behaviors was observed among the study sample, with findings demonstrating that impulsivity moderately differentiates between the groups "with" and "without" suicidal ideation. Traumatic life events and substance use were also associated with suicide ideation, while social support was established as a protective factor against it.

## Introduction

Suicidal behavior is the 15th leading cause of death worldwide, accounting for 1.4% of all deaths in 2012.^1^ Given these alarming numbers and the historic increase in suicidal behavior rates in many countries, the World Health Organization (WHO) has committed to direct efforts to reduce suicide rates by at least a third, by 2030.^2^ The situation in Brazil is no different – at least 0.84% of the 1,832,649 deaths recorded by the Brazilian Unified Health System (Sistema Único de Saúde [SUS]) in 2021 were attributed to self-inflicted injury; a number slightly lower than the global average, although also increasing progressively over the last 19 years.^2,3^

Distinguishing suicidal thoughts from suicide attempts, several studies have sought to investigate the risk factors and correlates linked to suicidal behavior, aiming to enrich prevention measures and facilitate clinical interventions.^4–7^ Analyzing this complex phenomenon according to gender, suicide rates are three times higher amongst men, with an even greater discrepancy observed in high-income countries.^1^ Regarding age, it was observed that suicide rates are higher among adults over 70 years old. However, this pattern reverses when death rates are disregarded, and when adults are investigated regarding "risk factors that surround suicidal behaviors" – showing a significant relationship between being female and expressing suicidal behavior and showing an inversely related relationship to age and educational level. In other words, as age and educational level decrease, the chances of suicidal behavior increase.^4^

Another frequently associated risk factor for suicidal behavior is the presence of previous mental health disorders.^4,5,8–11^ A meta-analysis investigated the relationship between the presence of mental disorders and suicide. This study demonstrated that among those with psychotic disorders, the risk ratio (RR) of committing suicide is approximately 13.2 times higher compared to individuals without psychotic disorders. Similarly, mood disorders, personality disorders, and substance use disorders have RRs of 12.3, 8.1, and 4.4 for suicide attempt, respectively.^12^ However, many disorders show significant differences in relative risk when analyzed by gender. For example, women with alcohol use disorder have a higher risk of suicide compared to men, whereas the situation is reversed with opioid use.^13,14^ Despite all this, it's important to point out that only 2 to 8% of individuals with different mental health conditions are at risk of suicide, while the majority of these individuals do not die by suicide.^5^

So far, the umbrella term "suicidal behavior" has been used to refer to a wide and complex range of observable behaviors, considering that most of the statistics for this phenomenon use this same umbrella term. However, it is important to stress some distinctions that need to be considered when using the term. For example, in the group of people with suicidal ideation, there are those who, in addition to thoughts of death, establish a plan on how to take their own life, and those who do not plan, only having a desire to commit suicide. This particular approach is relevant because the probability of an attempt is 56% in the first group with planning, compared to just 15.4% in the second group without planning.^11,15,16^ Similarly, when studying self-harm, it is important to differentiate "non-suicidal self-injury" (harming oneself without the intention of dying) from "suicidal self-directed violence" (harming oneself with the intention of dying); bearing in mind that these two situations differ in terms of function, severity, and method.^17–20^

There are several reasons why the distinction between suicidal ideation and attempt should be emphasized in scientific research. Amongst these, it must be considered that most of the risk elements predicting suicidal ideation do not show an equal relationship with suicide attempts.^8,10,21^ For example, Nock et al.^4^ conducted a cross-cultural study in 21 countries with 108,664 respondents, demonstrating that a wide variety of mental disorders increase the chances of experiencing suicidal thoughts. However, when controlling for "comorbidity" in the group of "ideators," only those disorders characterized by anxiety or lag in impulse control were considered predictive for the transition between mere thought to concrete action itself. From the same perspective, post-traumatic stress disorder seemed to distinguish well the "ideators" from those who actually attempt suicide, showing a moderate effect for the observed difference. Finally, depressive symptoms seemed to differ little between these two groups.^22^

The role of impulsivity in suicidal behavior also needs to be considered. Three main theoretical models have been proposed to explain suicide, each based on different facets of impulsivity.^23^ However, some recent meta-analyses aiming to clarify this association found a weak positive relationship between impulsivity and suicidal tendency, suggesting that the evidence is still scarce for the context at hand.^24,25^ Observing from another perspective, some authors have argued that this relationship is well-established, although acknowledging that the literature is still confusing and contradictory, largely due to problems of definition and conceptual separation between some terms.^26,27^ For example, while "behavioral impulsivity" seemed to show no relationship with suicidal ideation, "cognitive impulsivity" appeared to be a good predictor of belonging to the "ideation" group.^28^

Difficulties related to impulsive behavior have been extensively studied in the prison population. Several studies have demonstrated that high levels of impulsivity are associated with various challenges in this population. These include substance use,^29–31^ self-harm,^29,32^ aggressive behavior,^33^ risky sexual behavior,^34,35^ resistance to socio-educational treatment,^36,37^ and suicide.^38,39^

Brazil currently has the third largest incarcerated population in the world, behind only the United States and China.^40^ The most recent survey conducted by the National Secretariat of Criminal Policies (Secretaria Nacional de Políticas Penais [SENAPPEN]) showed that the frequency of suicide amongst the prison population, considering the total number of deaths, is much higher than the national average. According to this report, there were 913 deaths among prisoners during a 6-month period, with almost 10% of them occurring due to suicide.^3,41^ The same pattern has been found in other research around the world: suicide is more frequent in prisoners than in the general population.^42,43^

The WHO report on suicide prevention in prisons, written in partnership with the International Association for Suicide Prevention (IASP), argued that, for populations of individuals deprived of liberty, suicide may seem the only way out of a desperate situation – one in which they might have reached a state of total hopelessness and narrowing of their future positive perspectives.^44^ In summary, several factors associated with prison environments are related to suicide, such as single cell occupation, serving a life sentence, and absence of social visits; likewise, second-order factors are also related, such as substance misuse, mental health difficulties, and suicidal thoughts.^7,45^ However, this is still an area that requires further investigation, considering that understanding and identifying risk factors associated with suicidal behavior can contribute to prevention and identify demands for resource allocation in the prison system, and also considering that interventions in such spaces are naturally challenging.

The focus of this research was on assessing predictors of suicidal ideation and of suicide attempts in a sample of male prisoners who use psychoactive substances. Various factors were evaluated: sociodemographic variables (age, education, family support), life history (traumatic experiences), physical and mental health (substance use), and cognitive traits (impulsivity). Our main goal was therefore to enhance comprehension of factors linked to suicidal behavior in a sample of individuals deprived of liberty in Brazil, investigating, in particular, how impulsivity acts as a predictor of suicidal ideation and suicide attempts, and how vulnerability factors associated with life history are also relevant to this outcome.

## Method

### Design and participants

The present study adopted a cross-sectional approach with the purpose of investigating the predictors of suicidal ideation and suicide attempt in individuals deprived of liberty who use psychoactive substances. In this context, the relationships between traits of impulsivity, sociodemographic characteristics, substance use, and the occurrence of suicidal ideation and suicide attempt throughout life were analyzed.

A total of 174 male individuals, all deprived of liberty in the Penitenciária Estadual de Arroio dos Ratos (state penitentiary), state of Rio Grande do Sul, Brazil, participated in the research. Only individuals serving closed prison sentences between September 2022 and January 2023 (the data collection period) were included in the sample. It should be noted that all data were collected through face-to-face interviews with the inmates. Institutional data regarding the prisoners were not accessed.

The participant recruitment process was conducted through extensive dissemination of oral invitations to the convict population and only those who expressed verbal interest in participating were then included in the study. Following this initial step, a screening process was carried out with those prisoners who showed interest and, finally, individuals who had cognitive, visual, auditory, and/or motor difficulties were excluded from the study sample before analysis.

### Instruments

#### Sociodemographic measures and substance use

The Addiction Severity Index (ASI-6)^46,47^ is a semi-structured interview instrument widely used by clinicians and researchers to assess the severity of substance dependence. The ASI-6 subscales demonstrate internal consistency (Cronbach's alpha) ranging from 0.64 to 0.95. Although the complete scale takes from 45 to 90 minutes to answer, for the purposes of this research, we opted for a reduced version, considering it would be much more appropriate to the scope of the study.

#### Impulsivity

The Barratt Impulsiveness Scale (BIS-11)^48,49^ consists of a 30-item Likert scale designed to assess the three main factors of impulsive behavior. Cronbach's alpha for the complete scale was calculated at 0.79. However, after data collection, a substantial number of omissions were observed in the full version of the scale. As a strategy for data analysis, we opted for the BIS-Brief model,^50^ aiming to maintain the highest number of respondents and eliminating omitted responses. A validation study for the BIS-Brief, conducted with a sample of North Americans, demonstrated the scale had good sensitivity for detecting differences between clinical groups, presenting a Cronbach's alpha of 0.78 for this model.^50^

#### Suicidal behavior

Variables of suicidal ideation and suicide attempt were evaluated through self-report, along with other information collected using the adapted version of the ASI-6. The mental health section includes questions about suicidal ideation ("Have you ever had thoughts about killing yourself?": yes/no) and suicide attempts ("Have you ever tried to kill yourself?": yes/no).

### Ethical considerations

Participation in this research was voluntary. All participants received a copy of the Free and Informed Consent Form, which informed them of all their rights, such as full anonymity and the possibility of withdrawal from the study at any time. The project was approved by the Research Ethics Committee at the Universidade Federal do Rio Grande do Sul (UFRGS) (CAAE 59006822.6.0000.5334), in compliance with National Health Council (Conselho Nacional de Saúde) resolution no. 466/2012.

### Data analysis

The analyses were performed using SPSS (version 29.0.0.0). The prevalence of suicidal ideation, suicide attempts, and other sample characteristics were determined through descriptive analyses. Then, bivariate analyses were conducted to evaluate the relationships between each individual variable and the grouping variables suicidal ideation and suicide attempt using the chi-square test of association (χ²) and the independent *t* test. Variables that were related to suicidal ideation or attempted suicide in the bivariate analyses were then included in two different binary logistic regression models. Furthermore, two additional binary logistic regression analyses were then conducted to investigate the impact of use of psychoactive substances on the outcomes of suicidal ideation and suicide attempt. The effect size statistics were interpreted according to work by Durlak^51^ and Cohen.^52^

## Results

### Demographic characteristics of the sample

The mean age of study participants was 32.7 years (standard deviation [SD] = 9.31; lower: 18; upper: 57), and they reported using the following substances: alcohol, marijuana, cocaine, crack, cigarettes, and hallucinogens. Only six individuals did not meet criteria for substance use. The descriptive data of the sample, as well as the prevalences of suicidal thought and suicide attempt are presented in Table 1.

**Table 1:** Sociodemographic characteristics of the sampled prisoners (n = 174)

Characteristic		n (%)
Age, mean (SD)		32.7 (9.31)
	
Marital status		
	Living as married	72 (41.4)
	Married	38 (21.8)
	Never married	34 (19.5)
	Others	30 (17.2)
	
Education		
	Primary education	123 (71.5)
	Secondary education	42 (24.4)
	Higher education	7 (4.1)
	
Race†		
	Caucasian	94 (58.4)
	Black	42 (28.0)
	Light-skinned black	22 (13.7)
	
Has children?		117 (70.5)
Has someone to count on?		147 (85.5)
	
Current substance use‡		
	Alcohol	114 (66.7)
	
Lifetime substance use‡		
	Tobacco	139 (80.8)
	Cannabis	134 (77.9)
	Cocaine	120 (70.2)
	Crack	51 (30.0)
	Hallucinogens	47 (27.3)
	
Suicidal ideation		62 (36.7)
Suicide attempts		27 (16.0)
Conviction for attempted murder		38 (24.7)
Has ever been assaulted or abused?		57 (33.1)
Witnessed someone being killed or beaten?		87 (50.6)

SD = standard deviation.

† In Brazil, different terms are used to refer to individuals with dark and light skin among the Black population (for more information, refer to Osorio^53^).

‡ Only alcohol was assessed for current use; the other substances were evaluated for lifetime use.

### Impulsivity and suicidal ideation

An independent *t* test was conducted to examine the extent to which levels of impulsivity differed between those who reported suicidal ideation and those who did not. The assumption of homogeneity of variance was verified with the Levene test (p = 0.157). The normality of the data was verified with the Shapiro-Wilk test The result demonstrated that the assumption of normality was not confirmed (p = 0.042). Therefore, we opted to employ the bootstrap procedure (1,000 resamples; 95% bias-corrected and accelerated CI [95% BCa CI]) to correct deviations in normality of the distribution of the sample and differences in group sizes, as well as to achieve greater reliability of results.^54^

The results showed that in a sample of incarcerated individuals who use substances, those who reported suicidal ideation had statistically higher impulsivity scores (M = 18.60, SD = 4.86) than those who did not (M = 16.44, SD = 5.20). This difference, −2.15, 95% BCa CI −3.82 to −0.49], was significant, t_[140]_ = −2.387, p = 0.011. The effect size for the difference between the means was moderate (*d* = −0.42).

### Suicide attempts and impulsivity

An independent *t* test was conducted to investigate to what extent impulsivity levels differed between those who reported a suicide attempt during their lifetime (yes and no). The assumption of homogeneity of variance was verified by the Levene test (p = 0.245). The Shapiro-Wilk test demonstrated that the assumption of normality of the data was not confirmed (p = 0.024). Therefore, the bootstrapping procedure (1,000 re-samplings; 95% BCa CI) was employed to correct for deviations from the normal distribution of the sample and differences in sample sizes between the groups.^54^

The results showed that impulsivity scores were higher among those who reported a suicide attempt (M = 18.73, SD = 4.59), compared to those who did not report a suicide attempt (M = 16.93, SD = 5.23). However, this difference, −1.80 95% CI BCa (-4.11, 0.34), was not significant (t_[140]_ = −1.417, p = 0.118). The effect size for the difference between the means was small (*d* = −0.34).

### Sociodemographic predictors

A series of chi-square tests of independence were conducted to examine whether there were any associations between the different sociodemographic grouping variables found in the database and the presence of suicidal ideation (yes or no). The results showed significant associations between suicidal ideation and the following variables: Having someone to count on (χ²_[1]_ = 10.651, p = 0.001), having suffered abuse or aggression in the past (χ²_[1]_ = 29.062, p < 0.001) and having witnessed someone being killed or beaten (χ²_[1]_ = 20.810, p < 0.001) (see Table 2).

**Table 2:** Associations between demographic characteristics and suicidal behavior

			Suicidal ideation,		Suicide attempt,	
Characteristics		Total	n (%)	p-value	n (%)	p-value
Age						
	18-27	55	23 (41.8)	0.113	8 (14.5)	0.173
	28-37	69	26 (37.7)		11 (15.9)	
	38-47	31	12 (38.7)		8 (25.8)	
	48-57	14	1 (7.1)		0 (0.0)	
					
Education						
	Primary education	119	46 (38.7)	0.693	17 (14.3)	0.486
	Secondary education	41	13 (31.7)		8 (19.5)	
	Higher education	7	3 (42.9)		2 (28.6)	
					
Race						
	Black	44	15 (34.1)	0.749	17 (18.7)	0.703
	Caucasian	91	37 (40.7)		3 (13.6)	
	Light-skinned black	22	8 (36.4)		6 (13.6)	
					
Substance use						
	Alcohol	112	47 (42.0)	0.077	20 (17.9)	0.423
	Tobacco	134	57 (42.5)	0.004	23 (17.2)	0.481
	Marihuana	131	54 (41.1)	0.037	24 (18.3)	0.149
	Cocaine	116	53 (45.7)	< 0.001	23 (19.8)	0.058
	Crack	49	26 (53.1)	0.008	14 (28.6)	0.006
	Hallucinogens	44	19 (43.2)	0.333	9 (20.5)	0.368
					
Has someone to count on?						
	Yes	144	46 (31.9)	0.001	19 (13.2)	0.013
					
Has ever been assaulted or abused?						
	Yes	57	37 (64.9)	< 0.001	18 (31.6)	< 0.001
					
Witnessed someone being killed or beaten?						
	Yes	86	46 (53.5)	< 0.001	18 (20.9)	0.079
					
Conviction for attempted murder						
	Yes	53	14 (26.4)	0.822	7 (18.4)	0.449

### Model for suicidal ideation and suicide attempt

Subsequently, all variables with significant results in the chi-square test were included in a logistic regression model (enter method) for suicidal ideation, to verify how sociodemographic and clinical characteristics could constitute risk factors for the outcome. No violations of the assumption of collinearity were found for the selected variables. The model was statistically significant (χ²_[4]_ = 40.621, p < 0.001, Nagelkerke R² = 0.348), being able to correctly predict 79.3% of the cases. Likewise, a model for suicide attempt was also statistically significant (χ²_[4]_ = 12.2, p = 0.016, Nagalkerke R² = 0.152), being able to correctly predict 68.6% of cases. Table 3 shows the predictors included in each of the two models and the individual contribution of each predictor.

**Table 3:** Multivariate associations (ORs) between suicidal behavior, impulsivity, and demographic characteristics

Variables		z	p-value	OR	95%CI for OR	
					Lower	Upper
Suicidal ideation						
	Impulsivity	2.14	0.032	1.098	1.008	1.197
	Has someone to count on?	-2.09	0.037	0.281	0.085	0.925
	Has ever been attacked or abused?	1.94	0.053	2.403	0.989	5.837
	Witnessed someone being killed or beaten?	3.66	< 0.001	5.173	2.143	12.486
	Intercept	-2.60	0.009	0.085	-	-
					
Suicide attempt						
	Impulsivity	1.378	0.168	1.077	0.969	1.198
	Has someone to count on?	-1.831	0.067	0.312	0.089	1.085
	Has ever been attacked or abused?	1.424	0.155	2.270	0.734	7.024
	Witnessed someone being killed or beaten?	0.938	0.348	1.725	0.551	5.399
	Intercept	-2.634	0.008	0.054	-	-

95%CI = 95% confidence interval; OR = odds ratio.

### Suicidal behavior and substance use

A binary logistic regression analysis (enter method) was conducted to examine which substances would predict the outcome of suicidal ideation (yes or no) in the sample of inmates. No violations related to the assumption of collinearity were found for the selected variables. The investigated model was statistically significant (X²_[5]_ = 21.675, p < 0.001, Nagelkerke R² = 0.169), capable of correctly predicting 65.6% of cases. However, a model for attempted suicide was not statistically significant (χ²_[6]_ = 9.564, p = 0.144, Nagelkerke R² = 0.096) (see Table 4).

**Table 4:** Multivariate associations (ORs) between suicidal behavior and substance use

Variables		z	p-value	OR	95% CI for OR	
					Lower	Upper
Suicidal ideation						
	Alcohol	1.539	0.124	1.806	0.850	3.836
	Tobacco	2.067	0.039	3.309	1.063	10.293
	Marihuana	-0.483	0.629	0.764	0.256	2.276
	Crack	1.562	0.118	1.846	0.855	3.958
	Cocaine	2.042	0.041	2.678	1.040	6.897
	Hallucinogens	-0.272	0.786	0.895	0.403	1.988
	Intercept	-4.031	< 0.001	0.075	-	-
					
Suicide attempt						
	Alcohol	0.659	0.510	1.383	0.526	3.633
	Tobacco	-0.114	0.909	0.927	0.252	3.402
	Marihuana	0.185	0.853	1.152	0.272	5.164
	Crack	2.066	0.039	2.706	1.052	6.959
	Cocaine	0.745	0.456	1.634	0.448	5.950
	Hallucinogens	0.660	0.509	1.398	1.516	3.787
	Intercept	-3.474	< 0.001	0.063	-	-

95%CI = 95% confidence interval; OR = odds ratio.

Only alcohol was assessed for current use; the other substances were evaluated for lifetime use.

Next, a chi-square test of independence was performed to analyze the association between suicidal ideation (yes and no) and the frequency of use of illegal substances in general, considering the period of life with the highest use (zero to three times per month and one or more times per week). A significant association was found between suicidal ideation and the frequency of use of illegal substances (χ²_[1]_ = 6.673, p = 0.010, φ = 0.207). OR analyses showed that harmful use of illegal substances (one or more times per week) is associated with a 4.04 times greater chance of experiencing suicidal ideation at some point in life.

## Discussion

This study provides exploratory evidence on the role of impulsivity, substance use, and life history factors in the presence of suicidal ideation and suicide attempt among incarcerated men. Based on the data, it was possible to confirm the main hypothesis that the group with suicidal ideation would have higher impulsivity scores when compared to the non-suicidal ideation group. These results align with other studies in the literature, indicating that the presence or absence of suicidal ideation is associated with significant differences in impulsivity scores.^28,55–58^

However, these data should be interpreted with caution. In a meta-analytical study with 77 articles, Moore et al.^25^ provided evidence that suicidal tendencies in adults have a weak positive relationship with impulsivity. Other factors, such as life history and, in particular, experiences of negative events during development and/or adulthood, such as physical and sexual abuse, have strong relationships with suicidal behavior and may be more relevant for understanding the phenomenon.^59,60^ A similar pattern of association was found in our data: while impulsivity had a moderate effect size for distinguishing individuals with and without suicidal ideation, its effect was low as a predictor in the logistic analysis, while traumatic factors related to life history made greater contributions to the model.

Some studies suggest that suicidal behavior may vary depending on the type of trauma experienced throughout life.^8,61,62^ For example, suicide attempts were more strongly associated with childhood physical abuse, violent death of a loved one, and sexual abuse.^63^ Another study, based on epidemiological data, found higher frequencies of suicidal ideation associated with personal experiences such as childhood neglect and abuse (physical violence and sexual abuse), with moderate frequency related to witnessing someone severely injured/killed or finding a corpse.^64^ Our results align with these findings, confirming that suicidal behavior is related to past traumatic experiences. However, they differ regarding the impact of different forms of traumatic experience. In our sample of incarcerated men, the experience of witnessing someone being killed, assaulted, or beaten was a stronger predictor of suicidal ideation than experiencing some form of assault or abuse by an acquaintance during life – an association that was marginally significant in our context. It can be inferred that the response pattern found is due, in part, to a recency effect, in that the temporal sequence in which events occurred during individuals’ lives facilitates recall.^65^ Taking into account the high frequency of violent deaths amongst prisoners, it is expected that a considerable number of these individuals have witnessed hostile situations, making them particularly memorable due to their temporal proximity.^41,65^

Regarding the support received from friends and family, the results indicated that "having someone to rely on" is a protective factor against suicidal ideation. In the research sample, there was a 71.9% reduction in the odds of a fatal outcome for those who reported having someone to count on. However, mental health investigations have shown that this is not significantly associated with family support during the period of incarceration, but that "received support" is associated with mental health outcomes during reintegration into society.^66,67^ However, findings are controversial: some studies have demonstrated an association between family support and a reduced risk of smoking, of having sexually transmitted infections, and of experiencing malnutrition (the last of these studies was conducted in a country with high social vulnerability).^68–70^ Based on these contrasting data, it can be suggested that even if the support received is not a determinant of the mental health of inmates, it is a relevant protective factor for various physical and mental health conditions.

Similar to previous research,^6,63,71–73^ substance use was identified as a factor that increases the likelihood of suicidal ideation. Interestingly, and contrary to previous studies,^63,74–77^ current alcohol use was not significantly associated with suicidal ideation; only lifetime use of substances such as tobacco and cocaine showed a significant contribution to the outcome.^73^ Furthermore, it is important to note that the relationship between the study variables is likely bidirectional. Factors such as impulsivity, substance use, and mental health conditions have a reciprocal relationship, meaning both that increased substance use can worsen mental health conditions and vice versa.^63,78^

Contrary to our expectations, statistically significant differences in impulsivity levels were not found between those who reported suicide attempts and those who did not.^73,79^ Likewise, no significant results were found for the life history variables or for substance use in the regression analyses. It can be considered that the results found for the aforementioned analyses are due to the low sample size of people with attempted suicide. It is important to consider that works such as Nock et al.^11^ and Klonsky et al.^15^ provide evidence for the relationship between suicide attempts and impulse control disorders, post-traumatic stress, and substance use. In future studies focusing on individuals deprived of liberty, it is relevant to explore the association between impulsivity and suicide attempts using larger samples, considering sociodemographics and accounting for mental health predictors. It could thus be possible to clearly draw distinctions between the risk factors for suicidal ideation and attempted suicide, focusing on the population of individuals deprived of liberty – something that was not possible to achieve in this study.

There are limitations that need to be considered when interpreting the results presented in this study: first, neither suicidal behavior nor traumatic experiences throughout life were located in time in the analysis, which limits the conclusions that can be drawn from this relationship. Second, mood disorder variables, such as depression and anxiety, were not assessed; while there is evidence for the mediating role that psychiatric comorbidities play in the relationship between impulsivity and suicide attempts.^80^ Third, some results presented were only marginally significant and should not be taken as conclusive. Finally, impulsivity was treated as a unitary construct, making it difficult to measure the components of impulsivity more narrowly.^50^ Future studies conducted with inmates should consider a larger sample size for the detection of proposed effects, measuring suicidal ideation in conjunction with other related conditions in participants, as well as exploring conceptual distinctions within impulsivity and suicide.

## Conclusion

Within the population of incarcerated individuals who use psychoactive substances, those reporting suicidal thoughts had elevated impulsivity levels in comparison to those without such thoughts. However, membership in the "ideation group" is more strongly linked to experiencing traumatic events than levels of impulsivity. Furthermore, there are positive correlations between cigarette and cocaine use and suicidal thoughts. Conversely, the data indicate that having a reliable support system serves as a protective factor against suicidal behavior. These results underscore the significance of mental health care for the incarcerated, emphasizing potential focal points for intervention and prevention strategies that target suicidal behavior within confinement.

## Data Availability

The dataset collected and analyzed to produce this article is not yet publicly available. Other studies with the same dataset are still ongoing; however, specific data may be provided in response to custom requests.
